# Disgust and the rubber hand illusion: a registered replication report of Jalal, Krishnakumar, and Ramachandran (2015)

**DOI:** 10.1186/s41235-018-0101-z

**Published:** 2018-05-16

**Authors:** Hiroshi Nitta, Haruto Tomita, Yi Zhang, Xinxin Zhou, Yuki Yamada

**Affiliations:** 1 Graduate School of Human-Environment Studies, Kyushu University, 6-19-1 Hakozaki, Higashi-ku, Fukuoka, 812-8581 Japan; 2 Faculty of Arts and Science, Kyushu University, Fukuoka, Japan

**Keywords:** Disgust, Obsessive-compulsive disorder, Replication, Rubber hand illusion

## Abstract

Heightened experience of disgust is a feature of obsessive-compulsive disorder (OCD), particularly contamination-related OCD (C-OCD). Previous studies of the rubber hand illusion (RHI) reported that the sense of body ownership is related to the interaction between vision, touch, and proprioception. One recent study demonstrated a link between the RHI and disgust, suggesting that there is an interaction between these three perceptual modalities and disgust (Jalal et al., PLOS ONE 10:e0139159, 2015). However, there have been no direct replications of this initial study. We therefore performed a direct replication of Jalal et al.’s (PLOS ONE 10:e0139159, 2015) study. We examined 133 participants (based on a power analysis) to determine whether placing contamination-related stimuli on a rubber hand causes OCD-like disgust among healthy participants experiencing the RHI. That is, we tested whether Japanese participants experience more intense disgust when the rubber hand and the participant’s hidden hand are stroked synchronously than when stroked asynchronously, in order to replicate and examine the cross-cultural validity of this effect. The main finding of the original study by Jalal and colleagues was successfully replicated in a large sample. Some inconsistencies in one of the control procedures exploring coldness sensations during the RHI were found, which could possibly be due to cross-cultural differences or the improved statistical power of the present study. Based on the present replication study, we conclude that an intervention using the RHI as proposed by Jalal et al. (PLOS ONE 10:e0139159, 2015) might potentially be useful for the treatment of OCD following replications in clinical OCD populations.

**Preregistration details:** This study was preregistered with Cognitive Research: Principles and Implications. The Authors’ protocol received in-principle acceptance on 31 March 2017. The preregistered protocol is available here: 10.6084/m9.figshare.6217295.

## Significance

Obsessive-compulsive disorder (OCD) is a debilitating disorder that typically involves uncontrollable obsessive thoughts and compulsive behaviors. The heightened experience of disgust is an important component of OCD. Severe OCD symptoms significantly influence patients’ quality of life. However, there are few effective treatments available for OCD. In a recent study, Jalal, Krishnakumar, and Ramachandran (2015) reported that OCD-like contamination sensations were triggered by a disgust-related stimulus during the rubber hand illusion (RHI). Jalal et al. wanted specifically to explore whether the RHI could be used toward developing a novel treatment for OCD. Indeed, their findings raised the possibility that the RHI could be a useful approach in the clinical treatment of OCD. However, for the RHI to be useful in a clinical setting for the treatment of OCD, it would require a high level of reproducibility and a substantial effect size. We conducted a direct replication of Jalal et al.’s (2015) study to test the reliability of the original findings. In addition, our replication study tested the cross-cultural validity of the results—that is, whether they could be generalized to Japanese participants. We found that disgust-related stimuli on a rubber hand induced significantly higher disgust in participants during the RHI compared to the control condition. Based on the present findings, we can conclude that intervention using the RHI might potentially be useful for the treatment of OCD although replication in clinical OCD populations are needed.

## Introduction

Obsessive-compulsive disorder (OCD) is a debilitating condition that typically involves obsessive thoughts and compulsive behaviors that cannot be controlled, even if the patient recognizes them as symptoms of their disorder (Del Casale et al., 2011; Jalal et al., 2015; Wu et al., 2016). Severe OCD symptoms interfere with all aspects of life, including work, school, and social relationships.

The heightened experience of disgust is an important component of OCD, especially in contamination-related OCD (C-OCD). A number of studies have suggested a relationship between disgust and the symptoms of C-OCD, particularly the fear of contamination (Ludvik, Boschen, & Neumann, 2015; Tolin, Worhunsky, & Maltby, 2004; Whitton, Henry, & Grisham, 2014). For example, Tolin et al. (2004) reported that individuals with OCD maintained the belief that contamination is transferred between originally uncontaminated objects for longer than patients with other anxiety disorders or non-anxious control participants. This phenomenon has been referred to as “the law of contagion” (Tolin et al., 2004). Tolin et al. (2004) reported that C-OCD is associated with an increased prevalence of disgust-like cognitive responses. Furthermore, several previous studies of cognitive reasoning have reported that individuals with OCD experience a stronger sense of disgust and an increased likelihood of irrational fear of contamination and a risk of becoming ill (Cisler, Brady, Olatunji, & Lohr, 2010; Verwoerd, de Jong, Wessel, & van Hout, 2013).

Cisler et al. (2010) reported that obsessive beliefs, particularly the overestimation of threat, are related to a heightened level of disgust that increases fear of contamination. Similarly, Verwoerd et al. (2013) reported that participants who exhibited more contamination fear tended to exhibit more fearful responses to reading stories containing disgust-related content. In addition, the study found that participants were more likely to report a feeling that they would become ill after reading a script that described an actor experiencing disgust, compared with a script in which the actor did not feel disgusted. Importantly, this phenomenon has been reported to occur in situations with low levels of actual contamination threat. Taken together, these findings suggest the possibility of a link between OCD symptoms, disgust, and cognitive reasoning.

The results of several previous studies have indicated that the interaction between the overestimation of threat and the tendency to experience disgust increases the fear of contamination. Thus, these studies support the important role of disgust in C-OCD. In recent experiments, Jalal and Ramachandran (2013, 2017) reported that disgust sensations could be elicited in individuals with OCD traits solely by observing another individual touching a stimulus they consider disgusting. In addition, they found that watching others washing their own hands produced vicarious relief from disgust (Jalal & Ramachandran, 2013, 2017).

A recent review focusing on effective behavioral strategies for C-OCD has suggested that traditional methods of exposure treatment, including exposure and response prevention (ERP; Meyer, 1966), are not always effective for reducing the experience of disgust (Ludvik et al., 2015). Thus, additional research is required to further the understanding of exposure treatment methods in C-OCD.

Research on a perceptual illusion could potentially elucidate the mechanisms underlying the relationship between disgust and C-OCD (Jalal et al., 2015). The rubber hand illusion (RHI) is a well-studied illusion first reported by Botvinick and Cohen (1998). In this illusion, a participant’s own hand is hidden from view, while they are instructed to visually fixate on a rubber hand. When the rubber hand and the participant’s real hidden hand are stroked synchronously for a period of time, the participant typically begins to feel that the rubber hand becomes a part of their own body. Studies of the RHI have suggested that the sense of body ownership is related to multisensory interactions between vision, touch, and proprioception (Botvinick & Cohen, 1998; Capelari, Uribe, & Brasil-Neto, 2009; Costantini & Haggard, 2007). Thus, participants perceive the rubber hand as their own hidden hand because the brain and nervous system processes the rubber hand as if it is receiving the same sensory inputs that the hidden real hand feels (Capelari et al., 2009; Costantini & Haggard, 2007; Kilteni, Maselli, Kording, & Slater, 2015).

A large number of studies have demonstrated a relationship between the RHI and pain-related perception or threat (Armel & Ramachandran, 2003; Capelari et al., 2009; Ehrsson, Wiech, Weiskopf, Dolan, & Passingham, 2007; Ramachandran & Altschuler, 2009; Schlereth, Magerl, & Treede, 2001). However, to date there has been relatively little investigation of the relationship between the RHI and disgust. A recent study by Jalal et al. (2015) provided the first examination of this relationship, reporting that placing contamination-related stimuli on a rubber hand while participants experienced the RHI induced disgust (Jalal et al., 2015).

The findings of Jalal et al.’s (2015) study provided insight into the relationship between the RHI and disgust. The authors suggested that the use of contamination-related stimuli on the RHI might be relevant to the treatment of OCD. To the best of our knowledge, there have been no registered replication reports (RRR) of Jalal et al.’s (2015) initial study. Here we propose a direct replication of Jalal et al.’s (2015) reported procedure to confirm the finding that OCD-like disgust could be triggered by contamination-related stimuli in the RHI. In addition, we plan to test whether the phenomena are sensitive to cultural context, by conducting the study with a sample of Japanese participants. In line with Jalal et al.’s original findings, we hypothesize that Japanese participants, when observing a contamination-related stimulus placed on a rubber hand, will experience more intense disgust when the dummy and the participant’s hidden hand are stroked synchronously than when stroked asynchronously.

## Methods

### Ethics statement

The experiment was conducted according to the principles of the Declaration of Helsinki (2013). The ethics committee of Kyushu University has approved the protocol (approval number: 2016-002). Participants provided informed consent before participating in this study, with the understanding that they could stop at any time if they feel sick due to the disgust-inducing stimuli.

### Design

Following the design of Jalal et al. (2015), the main experiment involved two conditions: (1) a synchronous condition; and (2) an asynchronous condition.

During the induction of the RHI, the fingers of the rubber hand and the hidden real hand were stroked with two identical paintbrushes for 5 min. In the synchronous condition, strokes were applied to a rubber hand and the participant’s real hand synchronously. In contrast, in the asynchronous condition, the timing of the strokes applied to the rubber and the participant’s hand were asynchronous. As in the original study, synchronism (synchronous vs asynchronous) was a within-participant factor. An additional control procedure tested coldness sensations vis-a-vis the RHI and also with synchronism (synchronous vs asynchronous) as a within-participant factor. The disgustingness condition and the coldness condition were tested between two separate groups.

### Power analysis and participants

Importantly, Jalal et al.’s (2015) original study revealed that participants reported more intense disgust in response to a disgust-related stimulus in the synchronous condition compared with the asynchronous condition. However, the synchronism of stroking did not affect disgustingness ratings in a clean tissue stimulus control condition. Because there is no non-parametric method for analyzing two-way interactions between synchronism and stimulus type factors, we examined the original data to calculate the difference in disgustingness ratings between the disgust stimulus and a clean tissue conditions (i.e. the “D-T” diff.) for each observer in the synchronous and asynchronous conditions. We performed Wilcoxon signed rank tests on these data to provide a power analysis. The results revealed values of *Z* = 2.7 and *N* = 11, the same as the original statistics. Following Rosenthal and DiMatteo’s (2001) procedure, we computed Cohen’s *d* from the standard normal deviate and the sample size of the main experiment in Jalal et al.’s (2015) original study. The analysis revealed an effect size with Cohen’s *d* = 2.8. However, given that the contrast for the main comparison was between within-participant factors (i.e. synchronism: synchronous vs asynchronous; stimulus type: disgust stimulus vs clean tissue), it was more appropriate to examine Cohen’s *dz*., rather than Cohen’s *d*. According to Cohen (1988), Cohen’s *d* can be translated to *d*z. as follows: Cohen’s *d* = *d*z.√2. Applying Cohen’s (1988) method revealed an effect size of Cohen’s *d*z. = 1.98 with Jalal et al.’s (2015) original data. Although studies with small samples tend to overestimate true effect sizes, overestimation may decrease with subsequent replications, a phenomenon termed the “Winner’s Curse” (Button et al., 2013). Therefore, we applied a required power level of 0.95 and 30% of the effect size, with Cohen’s *dz*. = 0.594. Using G*Power 3.1 power analysis software (Faul, Erdfelder, Buchner, & Lang, 2009), we computed the required sample size for the disgustingness condition as a function of effect size *d*z. = 0.594, the required significance level α = 0.05 and the required power level 1-β = 0.95. These calculations resulted in a sample size for the disgustingness condition of *N* = 41. Furthermore, considering the survival rate of 79% for the main experiment in the original study, the present study required at least 52 participants in the disgustingness condition. In addition, to match the ratio between the number of participants in the control experiment and the main experiment in the original study (18 to 14 participants), the minimum sample size for the coldness condition in the present study was *N* = 67. Therefore, it was necessary to recruit more than 52 participants for the disgustingness condition and at least 67 participants for the coldness condition. Data collection did not exceed 150 participants. The sample consisted of undergraduate and graduate students at Kyushu University. In addition, we matched the gender distribution and age range of the original study. Thus, 71% of participants in the disgustingness condition and 44% of participants in the coldness condition were female, with an age range of 18–25 years. All participants were Japanese.

### Apparatus and materials

Participants were tested individually in two rooms at Kyushu University with natural light. The disgustingness and coldness conditions were tested in separate rooms.

**Fig. 1:**
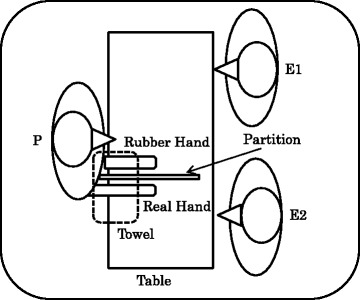
The experimental setup for the RHI

Participants were seated upright, resting their right arm with the palm down. A brown standing sagittal partition (approximately 45 × 65 cm) was positioned so that the participants could see only the rubber hand while their own right hand was concealed from view (i.e. the participants’ right arm was placed on the right side of the partition and the rubber hand was placed on the opposite side). The wrist of the rubber hand was wrapped with a colored towel (Fig. 1).

The experiment involves three fake disgust-related stimuli: (1) feces; (2) vomit; and (3) blood. After consulting with the first author of the original paper, Baland Jalal, we prepared the three disgust stimuli using commercially available items, based on information available online. The recipes for producing the disgust stimuli were obtained from two web sites: (1) feces (Uncyclopedia, 2013); and (2) vomit (Taiyaki, 2013). We bought a commercially available fake blood as (3) the blood stimulus.

To produce realistic odors for the feces and vomit stimuli, we used a commercially available product with a fecal odor and created a vomit-like odor using a recipe available online (Roketto Nyusu 24, 2010).

In the coldness condition, an ice cube was placed on the rubber hand, while an eraser shaped similarly to an ice cube is concurrently placed on the hidden real hand.

### Procedure

We directly replicated the procedure described in the original study (see Jalal et al., 2015). Participants were given a brief overview of the study and provided informed consent indicating their willingness to participate.

Each participant in the disgustingness and coldness conditions experienced both the synchronous and asynchronous conditions. The order of presentation of the two sub-conditions was counterbalanced across participants.

At the beginning of a trial in the disgustingness condition, participants were visually presented with each of the three stimuli at a visual distance of approximately 20 cm for 10 s. Participants then provided subjective ratings to indicate the level of disgust they felt in response to each of the three stimuli on a 20-point Likert-scale, with higher scores indicating greater disgust. For each participant, the disgust stimulus with the highest score was used in the disgustingness condition.

During the induction of the RHI, participants were instructed to fixate their gaze on the fingers of the rubber hand while the rubber hand and the participants’ hidden hand were stroked synchronously during the synchronous condition (or asynchronously in the asynchronous condition) with two identical paintbrushes for 5 min. Participants were required to verbally inform the experimenters as soon as they felt that the rubber hand was their own hand. Once participants indicated the onset of the RHI, the first experimenter immediately asked the following question: “どのくらい強く錯覚を感じますか” (How intense is the illusion?). Participants then rated the intensity of the RHI on a Likert-scale in the range of 1–20, with higher scores indicating greater intensity. After continuously stroking the rubber hand and the participant’s real hidden hand for 5 min, the second experimenter concurrently placed the disgust stimulus on the rubber hand and a clean tissue or a bandage on the participants’ real hand. If the disgust-related stimulus was feces or vomit, tissue paper with the stimulus was placed on the rubber hand, while a clean tissue moistened with water was placed on the real hand. If the disgust-related stimulus was blood, a bandage with a small amount of fake blood was placed on the rubber hand, while a clean damp tissue with a similar shape to the bandage was placed on the real hand for 15 s. Participants were asked the following question: “嫌悪感はどのくらいですか” (How disgusted do you feel?). Participants’ subjective ratings of disgustingness were assessed using a 20-point Likert-scale, with higher scores indicating greater disgustingness. Immediately after the disgustingness ratings, the disgust stimuli and the clean tissue were removed from each hand. Stroking of the rubber hand and the participants’ real hand continued for an additional 1-min period, to maintain the RHI.

The last phase of the disgustingness condition was a clean tissue control condition. In this condition, a clean tissue was simultaneously placed on the rubber hand and the participant’s own hidden hand for 15 s. Participants were asked to rate the disgustingness of the clean tissue just after the tissue had been placed on both the rubber and their hidden hands. In the inter-session period between the synchronous and asynchronous conditions, participants were instructed to perform a set of basic arithmetic exercises using their fingers with their eyes closed for approximately 2.5 min, to control for potential carry-over effects of the RHI. Following the inter-session period, participants performed the second condition.

After completing trials in both sub-conditions, participants were asked to report in which sub-condition they felt more intense illusion and to rate the intensity in that condition on a 20-point Likert-scale, with higher scores indicating greater intensity of the illusion. At the end of the experiment, participants were asked to describe what they thought the study was about. Participants were fully debriefed and thanked for their time.

It should be noted that the asynchronous condition was identical to the synchronous condition, except that strokes were applied to the rubber hand and the participant’s real hand asynchronously. Thus, participants were asked to report the onset of the RHI, to assess the intensity of the RHI in response to the question: “どのくらい強く錯覚を感じますか” (How intense is the illusion?), and also underwent the clean tissue control condition.

In the coldness condition, the apparatus and procedure were identical to the disgustingness condition, except for the following changes: (1) an ice cube was placed on the rubber hand instead of the three disgust stimuli and a rectangular-shaped eraser was placed on the hidden real hand instead of the clean tissue paper or the bandage; (2) participants performed the basic arithmetic exercises immediately after reporting subjective coldness when the ice cube was placed on the rubber hand and the rectangular-shaped eraser was placed on the hidden real hand. Thus, in the coldness condition, participants did not undergo the clean tissue control condition. Participants in the coldness condition were asked the following question: “冷たさはどのくらいですか” (How cold do you feel?), providing subjective ratings of coldness on a 20-point Likert-scale with higher scores indicating greater “coldness.”

### Data analyses

In the original study, non-parametric Wilcoxon signed rank tests were used to compare differences in disgustingness ratings in response to disgust stimuli between synchronous and asynchronous conditions. However, these data would also be suitable for analysis using two-way ANOVA to examine disgustingness ratings, with synchronism (synchronous vs asynchronous) and stimulus type (disgust stimulus vs clean tissue) as within-participant factors, provided the assumption of normality was met. According to the original study, however, the data clearly violated the assumption of normality. For this reason, we sought to compute the interaction effects between synchronism and stimulus type factors using non-parametric methods. However, none of these methods was able to provide an appropriate analysis. Therefore, we compared D-T diff.s in the synchronous and asynchronous conditions with an alternative method, using Wilcoxon signed rank tests to analyze comparisons. In addition, in the original study, the clean tissue control was performed only in the main experiment (not in the control experiment) and the control experiment did not measure disgustingness ratings. Thus, we analyzed the control condition using *t*-tests (or Wilcoxon signed rank tests) separately from the disgustingness condition in this study.

Significance tests for our replication data were based on the assumption of normality of the data. If all four conditions (i.e. disgust-synchronous, tissue-synchronous, disgust-asynchronous, and tissue-asynchronous conditions) passed the Kolmogorov–Smirnov test, a two-way ANOVA was appropriate for analyzing the disgustingness ratings with synchronism and stimulus type as within-participant factors. However, if any of the conditions did not pass the Kolmogorov–Smirnov test, Wilcoxon signed rank tests were more appropriate for analyzing D-T diff.s in the synchronous and asynchronous conditions. In contrast, coldness ratings in the coldness condition were most appropriately analyzed using paired *t*-tests or Wilcoxon signed rank tests to analyze comparisons between the synchronous and asynchronous conditions, based on the Kolmogorov–Smirnov test. Two-tailed *p* values were reported for all comparisons. In addition, as well as the conventional analyses described above, we used Bayes factors to compare the null and alternative hypotheses (Dienes, 2014). Table 1 lists all of the variables in the disgustingness condition and Table 2 lists all of the variables in the coldness condition. Moreover, Table 3 provides an overview of the analyses for each comparison.

**Table 1:** All of the variables in the disgustingness condition

Variables	Independent/Dependent
Synchronism (synchronous vs asynchronous)	Independent
The order of presentation (which sub-condition was completed first)	Independent
Stimulus type (disgust stimulus vs clean tissue)	Independent
Disgustingness rating	Dependent
Intensity of the RHI	Dependent
Intensity of the RHI on the condition where the RHI was more intense	Dependent

**Table 2:** All of the variables in the coldness condition

Variables	Independent/Dependent
Synchronism (synchronous vs asynchronous)	Independent
Coldness rating with an ice cube	Dependent
Intensity of the RHI	Dependent
Intensity of the RHI on the condition where the RHI more intense	Dependent

**Table 3:** Overview of the analyses for each comparison

What is compared	Variables	Type of analysis
Comparison of disgustingness ratings based on the order of presentation	IV: order of presentation DV: disgustingness rating	Paired *t*-test/Wilcoxon signed rank test
The difference of disgustingness ratings with a disgust stimulus and with a clean tissue during the synchronous vs asynchronous condition	IV: stimulus type/synchronism DV: disgustingness rating	A two-way ANOVA/Wilcoxon signed rank test
Comparison of coldness ratings during the synchronous and asynchronous conditions	IV: synchronism DV: coldness rating	Paired *t*-test/Wilcoxon signed rank test
Comparison of the mean intensity of the RHI between the disgustingness and coldness conditions	IV: condition DV: mean intensity	Independent samples *t*-test/Mann–Whitney U-test

*IV* independent variable, *DV* dependent variable

### Data exclusion criteria

As in the original study, we adopted the following exclusion criteria: (1) failure to complete all tasks properly or to provide adequate data; (2) reporting an experience of a more intense illusion during the asynchronous condition compared with the synchronous condition; and (3) reporting a score of < 3 out of 20 on the intensity of the RHI scale in the synchronous condition. All exclusion criteria were determined before the start of data collection.

## Results

As stated above, we initially performed the same analyses as Jalal et al. (2015) did in their study. In the present study, the data in all the conditions failed the Kolmogorov–Smirnov test. Because of this violation of the assumption of normality, a non-parametric Bayesian method was considered as a suitable alternative for the analysis. The non-parametric Bayesian model described by Gershman and Blei (2012) is broadly referred to as an underlying model for learning theory (Gershman, Pouncy, & Gweon, 2017; Griffin & Li, 2016; Littman, 2015) and as a non-parametric analog of factor analysis (e.g. Larsen, Hershfield, Stastny, & Hester, 2016). However, a non-parametric Bayesian analog for *t*-tests is not yet available as a statistical method (de Haan et al., 2017). Therefore, in accordance with the preregistered Bayesian analysis, here we reported the Bayes factors for the comparison of the mean D-T diff. scores during the synchronous and asynchronous conditions. We used the open-source software JASP (JASP Team, 2016; jasp-stats.org) to carry out the Bayesian analysis. JASP (version 0.8 Beta 5) gives us graphical user interface to easily interpret results with Bayesian statistics (Marsman & Wagenmakers, 2017; Quintana & Williams, 2017). In addition, we stated that we used the Wilcoxon signed rank test in the “Methods” section at the preregistration stage to compare the mean D-T diff. ratings between the different presentation orders of the synchronism conditions (i.e. the order in which the synchronous and asynchronous conditions were carried out) in the disgustingness condition. However, considering that the order of presentation was a between-subject factor, the Mann–Whitney U-test was deemed more suitable for the comparisons, so we used it as a correction of the pre-registered analysis. Moreover, we used the effect size *r* for the non-parametric test.

### Unregistered analysis: participant characteristics of Jalal et al.’s (2015) study and the present replication study

Because the data did not pass the Kolmogorov-Smirnov test (*Ds* = 1.00, *ps* < 0.0001), we compared the demographic data of Jalal et al. (2015) and our replication study using the Mann–Whitney U-test. No significant differences were found in age between the studies for both comparisons (main vs disgustingness conditions: *z* = 0.46, *p* = 0.64, *r* = 0.06; control vs coldness conditions: *z* = 0.62, *p* = 0.54, *r* = 0.07). The demographic characteristics and the results of the statistical comparisons between conditions are shown in Table 4. In Table 5, we describe the characteristics of the disgust stimuli used in the experiment.

**Table 4:** Participant characteristics of Jalal et al. (2015) and the present study

		Jalal et al. (2015) main condition					Our disgustingness condition					*z*	*p*	*r* effect size
		*N*	%	*Mean (SE)*	*SD*	Range	*N*	%	*Mean (SE)*	*SD*	Range			
All participants^a^	Gender	14		21.00 (0.55)	2.08	18–25	61		21.23 (0.24)	1.85	18–25	0.46	0.64	0.06
	Female	10	71				43	71						
	Male	4	29				18	29						
Final sample	Gender	11		21.20 (0.64)	2.14	18–25	52		21.35 (0.25)	1.82	18–25			
	Female	8	73				37	71						
	Male	3	27				15	29						
		Jalal et al. (2015) control condition					Our coldness condition					*z*	*p*	*r* effect size
		*N*	%	*Mean (SE)*	*SD*	Range	*N*	%	*Mean (SE)*	*SD*	Range			
All participants	Gender	18		21.17 (0.37)	1.58	18–25	72		21.47 (0.23)	1.96	18–25	0.62	0.54	0.07
	Female	8	44				30	42						
	Male	10	56				42	58						
Final sample	Gender	12		21.00 (0.51)	1.76	18–25	67		21.45 (0.24)	1.98	18–25			
	Female	5	42				29	43						
	Male	7	58				38	57						

^a^“All participants” included participants who were excluded from the final analyses

**Table 5:** Participants’ disgust stimuli

*N* = 52	*N* (%)	*M* (*SD*)
Feces	28 (54%)	15.68 (3.40)
Vomit	20 (38%)	15.75 (3.14)
Blood	4 (8%)	19.50 (1.00)
Total	52 (100)	16.00 (3.30)

The number (and proportions) of each stimulus selected as the most disgusting stimulus and the mean scores (and standard deviations) of disgustingness. When a participant rated the stimuli (e.g. “vomit” and “feces”) equally, an experimenter subsequently asked the participant to judge which stimulus was more disgusting; the stimulus judged to be more disgusting was used as the participant’s stimulus for the experiment

### Preregistered analysis: randomization of presentation

**Fig. 2:**
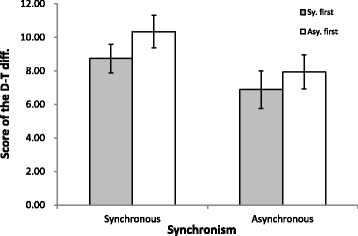
Mean D-T diff. scores during the synchronous and asynchronous conditions for each order of presentation. *Error bars* represent the standard error of the mean

Table 6 presents the mean scores and standard errors for D-T diff. during the synchronous and asynchronous conditions for each order of presentation (*N* = 52) in the disgustingness condition. None of the D-T diff. data passed the Kolmogorov–Smirnov test (*Ds* > 0.86, *ps* < 0.0001), so we compared the order of presentation (synchronous condition first vs asynchronous condition first) of the synchronous and asynchronous conditions for the disgustingness ratings using the Mann–Whitney U-test. Consistent with the findings of Jalal et al.’s (2015) study, there was no significant difference in the order of presentation (synchronous condition: *z* = 0.98, *p* = 0.32, *r* = 0.19; asynchronous condition: *z* = 0.86, *p* = 0.38, *r* = 0.17; Fig. 2). The effect sizes in the original article (synchronous condition: *r* = 0.51; asynchronous condition: *r* = 0.48) are relatively higher respectively compared to those in the present replication.

**Table 6:** The effect of order of presentation

Disgustingness		
	Synchronous first	Asynchronous first
Synchronous	8.73 (0.85)	10.35 (1.12)
Asynchronous	6.88 (0.97)	7.92 (1.02)

Mean scores (and standard errors) for D-T diff. during the synchronous and asynchronous conditions for each order of presentation are shown. “Synchronous first” denotes that participants experienced the synchronous condition first; “Asynchronous first” denotes that participants experienced the asynchronous condition first

### Preregistered analysis: disgustingness during the synchronous and asynchronous conditions

**Fig. 3:**
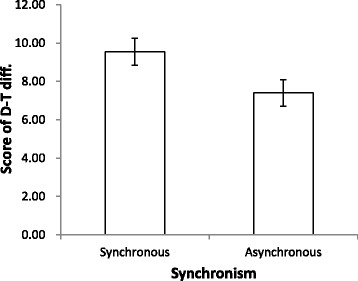
Mean D-T diff. scores during the synchronous and asynchronous conditions. *Error bars* represent the standard error of the mean

The final sample of the disgustingness condition was 52 participants; nine additional participants were excluded from the final analysis because one failed to complete all the tasks; six had a more intense RHI during the asynchronous stroking condition; and two, during synchronous stroking, had a score of < 3 out of 20 on the scale measuring the intensity of the RHI. Mean scores (and standard errors) for disgustingness ratings and D-T diff.s during the synchronous and asynchronous conditions are presented in Table 7. Because the D-T diff. scores in all four conditions (i.e. disgust-synchronous, tissue-synchronous, disgust-asynchronous, and tissue-asynchronous) failed the Kolmogorov–Smirnov test (*D* > 0.84, *ps* < 0.0001), we used the Wilcoxon signed rank test for comparisons. We observed a significant difference between the conditions, *z* = 4.09, *p* < 0.001, *r* = 0.57. Participants reported more intense disgust during the synchronous condition than during the asynchronous condition (Fig. 3). This is broadly consistent with Jalal et al.’s (2015) findings that nine out of 11 participants reported experiencing more intense disgust during the synchronous condition in comparison to the asynchronous condition, with *r* = 0.81.

**Table 7:** The results of the disgustingness condition

	Synchronous		Asynchronous	
	Stimuli	Tissue	Stimuli	Tissue
Rating	10.96 (0.74)	1.42 (0.16)	8.67 (0.72)	1.27 (0.11)
D-T diff.	9.54 (0.71)		7.40 (0.70)	

Mean scores (and standard errors) for the disgustingness ratings and D-T diff. scores during the synchronous and asynchronous conditions in the disgustingness condition

We additionally performed a Bayesian analysis to evaluate the null hypothesis and the alternative hypothesis for the main finding. For our Bayesian *t*-test alternative, we compared two models for effect size δ: the null hypothesis that the effect sizes of the mean D-T diff. rating during the synchronous and asynchronous conditions are equal (δ = 0) and the alternative hypothesis that the mean D-T diff. during both conditions are different. Based on the previous results shown by Jalal et al. (2015), we anticipated that the mean D-T diff. during the synchronous condition was higher than during the asynchronous condition. This previous result suggests the one-sided hypothesis H_+_: Mean T-D diff. during the synchronous condition > Mean T-D diff. during the asynchronous condition. We calculated the effect size of the original research with the data the original study reported and then we modified the effect size considering the overestimation of true effect size. We finally settled the effect size of the original research was *dz*. = 0.594. By integrating this prior information, we assigned δ a zero-centered Cauchy distribution prior, with an interquartile range *r* = 0.594 [δ~ Cauchy(0, 0.594)] (i.e. 50% of the prior mass falls in the interval from – 0.594 to + 0.594). The results indicated that the data were more likely to occur under the one-sided hypothesis (BF_+_ = 2209.88), with a 95% credible interval range of 0.324–0.920. A Bayes factor > 150 is taken as substantial evidence in favor of the alternative hypothesis (Kass & Raftery, 1995). Thus, the results in the present study showed that the present study successfully replicated the main findings of Jalal et al. (2015).

### Preregistered analysis: coldness ratings in the synchronous and asynchronous conditions

**Fig. 4:**
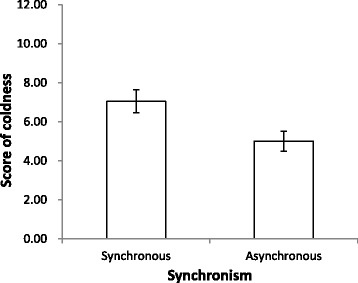
Mean scores for participants’ coldness ratings during the synchronous and asynchronous conditions. *Error bars* represent the standard error of the mean

The distribution of coldness ratings (*N* = 67) during the synchronous and asynchronous conditions in the coldness condition also failed the Kolmogorov–Smirnov test (*Ds* = 0.84, *ps* < 0.0001). Five additional participants were excluded from the analysis because three had a more intense RHI during the asynchronous stroking and two, during synchronous stroking, had a score of < 3 out of 20 on the scale measuring the intensity of the RHI. In contrast to Jalal et al. (2015), we found that there was a significant difference in the coldness ratings between the synchronous and asynchronous conditions (*M* = 7.07, *SD* = 4.84 vs *M* = 4.91, *SD* = 4.17), *z* = 4.93, *p* < 0.001, *r* = 0.60 (Fig. 4). In contrast, Jalal et al. (2015) found no significant difference between the mean scores for participants’ coldness ratings during the synchronous and asynchronous conditions, with *r* = 0.04 in the original study. We discuss this inconsistency in the “Discussion” section.

### Preregistered analysis: comparison of the mean scores on the intensity of the RHI scale during the synchronous condition in the disgustingness and coldness conditions

**Fig. 5:**
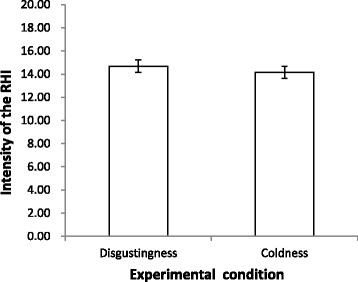
Participants’ mean scores on the intensity of the RHI scale. The scores during the synchronous condition in the disgustingness and coldness conditions are plotted. *Error bars* represent the standard error of the mean

To ensure that there was no difference between the intensity of the RHI during the synchronous condition in the disgustingness and coldness conditions,  we compared the mean intensity scores between the disgustingness and coldness conditions. We again conducted the Kolmogorov–Smirnov test and confirmed that the distribution of scores in both the disgustingness and coldness conditions (*N* = 119) were non-normal (*Ds* = 1.00, *ps* < 0.0001). A Mann–Whitney U-test revealed no significant difference in the mean score on the intensity of the RHI scale in the synchronous condition between the disgustingness and coldness conditions (*M* = 14.69, *SD* = 3.84 vs *M* = 14.15, *SD* = 4.38), *z* = 0.45, *p* = 0.65, *r* = 0.06 (Fig. 5). This is somewhat lower than the effect size found in Jalal et al. (2015), with *r* = 0.27 in the original article.

## Discussion

The main goal of this study was to replicate the findings of Jalal et al.’s (2015) study using a Japanese sample. The results of the present study demonstrated that placing disgust-related stimuli on a rubber hand did in fact induce stronger OCD-like disgust reactions during the RHI as compared to the control condition (asynchronous stroking of the dummy). This supports the hypothesis proposed by Jalal et al. (2015) that, in this case, a sense of body ownership occurs as a four-way multisensory interaction between vision, touch, proprioception, and disgust. The results of the present study also provided evidence of the cross-cultural validity of the effect of the RHI on increased feelings of disgust—that is, we found that the relationship between the RHI and disgust is robust in a Japanese sample. Cross-cultural validity is important to consider because Asians have a stronger tendency to feel OCD-like disgust than do White and Black people (for review, Wu & Wyman, 2016). In addition, as Curtis, de Barra, and Aunger (2011) argued, information related to pollution shared by a particular culture may be attributed to variations in disgust proneness.

ERP treatment is regarded as the primary non-pharmacological treatment for C-OCD (Jalal & Ramachandran, 2017; Mancebo, Steketee, Muroff, Rasmussen, & Zlotnick, 2017), but several studies have shown that the conventional ERP does not always work effectively for C-OCD (Ludvik et al., 2015; Widen & Olatunji, 2016). As Jalal et al. (2015) originally demonstrated and the present study confirmed, participants under the RHI felt disgust toward contamination-related stimuli without actually being in direct (skin-to-skin) contact with these stimuli. These findings suggest that the RHI could potentially be incorporated into treatments for OCD.

As for the coldness ratings, our results are not consistent with the results of Jalal et al.’s (2015) study. We suggest some possible explanations for this discrepancy. One is that the inconsistencies derive from differences in the sample characteristics between the studies. This replication study sampled Japanese individuals, whereas Jalal et al.’s (2015) study used Caucasians. Our results suggest that Japanese people might be more sensitive to cold stimuli placed on the rubber hand during the RHI, suggesting that there are cultural differences in coldness perception. Although many studies have examined the relationship between the RHI and cold pain (e.g. Mohan et al., 2012; Siedlecka, Klimza, Łukowska, & Wierzchoń, 2014), only a few have investigated the relationship between the RHI and cold perception itself. One past study has demonstrated that the skin temperature of a participant’s hand decreased during the RHI (Moseley et al., 2008). However, a recent replication study by de Haan et al. (2017) failed to support the results of Moseley et al. (2008). Therefore, much remains unknown about the link between the RHI and temperature sensation, or about cultural differences in sensitivity to cold stimuli during the RHI.

A potential alternative explanation for the discrepancy is a response bias specific to Japanese individuals. Kondo, Saito, Deguchi, Hirayama, and Acar (2010) found that Japanese individuals tend to answer in the same way as the majority even when the majority’s answer is not socially acceptable. In other words, the study indicated that Japanese people are prone to social desirability bias by following the majority’s opinion. Based on Kondo et al.’s (2010) findings, in the coldness condition, participants might have reported feeling cold during the synchronous condition more than during the asynchronous condition in order to please the experimenters, even if they did not actually feel cold. Can this bias explain the discrepancy? We believe that it cannot. Were it so, the Japanese sample would have likely provided positive answers during the asynchronous condition as well (i.e. they would have had similar cold ratings as for the synchronous condition); however, the findings showed that most participants actually reported “1” during the asynchronous condition (i.e. they did not feel cold at all). In addition, all the participants were naive to the purpose of the present study. Thus, we concluded that the significant difference in the reported cold scores between in the synchronous and asynchronous conditions was not due to the response bias.

Moreover, we need to consider the statistical power. The present study had more statistical power and thus was able to detect actual differences in coldness sensations, whereas Jalal et al. (2015) relied on a smaller sample and thus they could not. However, note that the fact that coldness sensations (in addition to disgust) arise from the dummy during the RHI does not negate the key “disgust” findings; it potentially shows the strength and versatility of the illusion. In a future study, it would be important to investigate the differences in the relationship between coldness perception and the RHI among various cultural samples. The present study was the first to replicate Jalal et al. (2015); as such, at this time, we cannot directly compare our findings with other replication studies to examine the cause of this discrepancy.

We also need to keep in mind one limitation of our methodology in the present study. Before the experiment, participants in the disgustingness condition were presented with the three disgust-related stimuli (i.e. vomit, feces, and blood) at a visual distance of approximately 20 cm and asked to report their level of disgust for each stimulus; the stimulus they judged as most disgusting was used for the experiment. During the experiment, however, participants observed the stimulus at a visual distance of further than 20 cm. This difference in physical distance of stimulus presentation might lead to a decrement in the emotional intensity of the disgust stimuli during the experiment. Indeed, some participants reported feeling more intense disgust during the initial presentation of the stimuli before the experiment compared to during the synchronous and asynchronous conditions because of the difference in distance. However, these self-reports may only show strong reactions due to the exposure to disgust stimuli for the first time. At least, in future studies it would be better to equalize the distance of the presentation during the stimulus selection process and the experiment as much as possible and further consider how the distance of disgust-related stimuli influences actual feelings of disgust under the RHI.

## Conclusion

In conclusion, the present study was the first replication attempt of Jalal et al. (2015). It closely followed their experimental design and analytic methods. We found that our Japanese sample experienced more intense disgust under the RHI compared to the control condition. This not only replicates but also supports the cross-cultural validity of these findings. As proposed by Jalal et al. (2015), the current procedure could pave the way for a novel treatment for OCD using the RHI. These results should be extended to a clinical OCD population in order to more directly explore their clinical utility. Moreover, future studies should examine the RHI vis-a-vis coldness sensations given the discrepancies in the literature.

## Abbreviations

C-OCD: Contamination-related OCD
ERP: Exposure and response prevention
OCD: Obsessive-compulsive disorder
RHI: Rubber hand illusion
